# Supplementary material for: Characterization of the O-Glycoproteome of Porphyromonas gingivalis
Source: Microbiol Spectr. 2022 Jan 5;10(1):e01502-21. doi: 10.1128/spectrum.01502-21 (PMC8729774; doi:10.1128/spectrum.01502-21)
Supplement: SUPPLEMENTAL FILE 1 — Supplemental material. Download SPECTRUM01502-21_Supp_1_seq13.pdf, PDF file, 0.3 MB [file spectrum01502-21_supp_1_seq13.pdf]

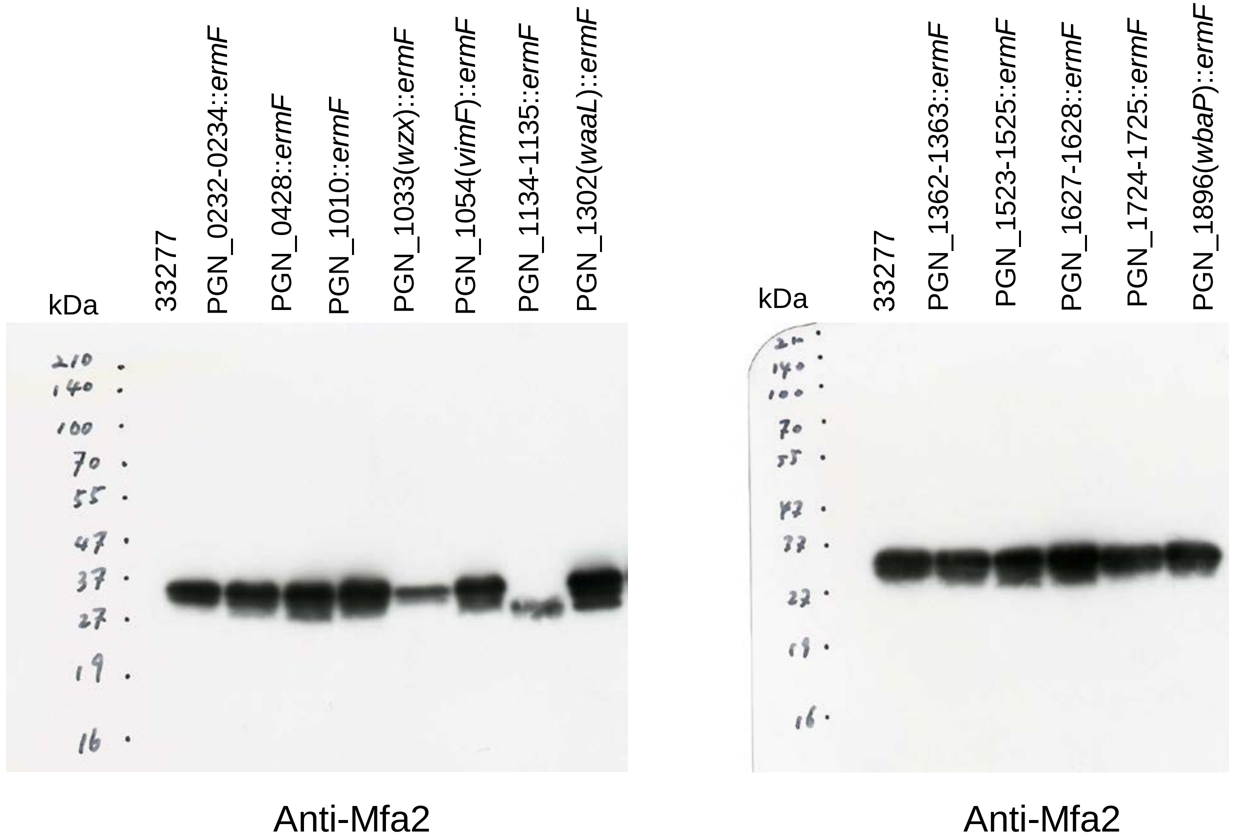

Figure S1. Western blot of various mutants against the Mfa2 protein. Whole cell lysates of the indicated strain were subjected to immunoblotting with polyclonal antibodies against Mfa2.

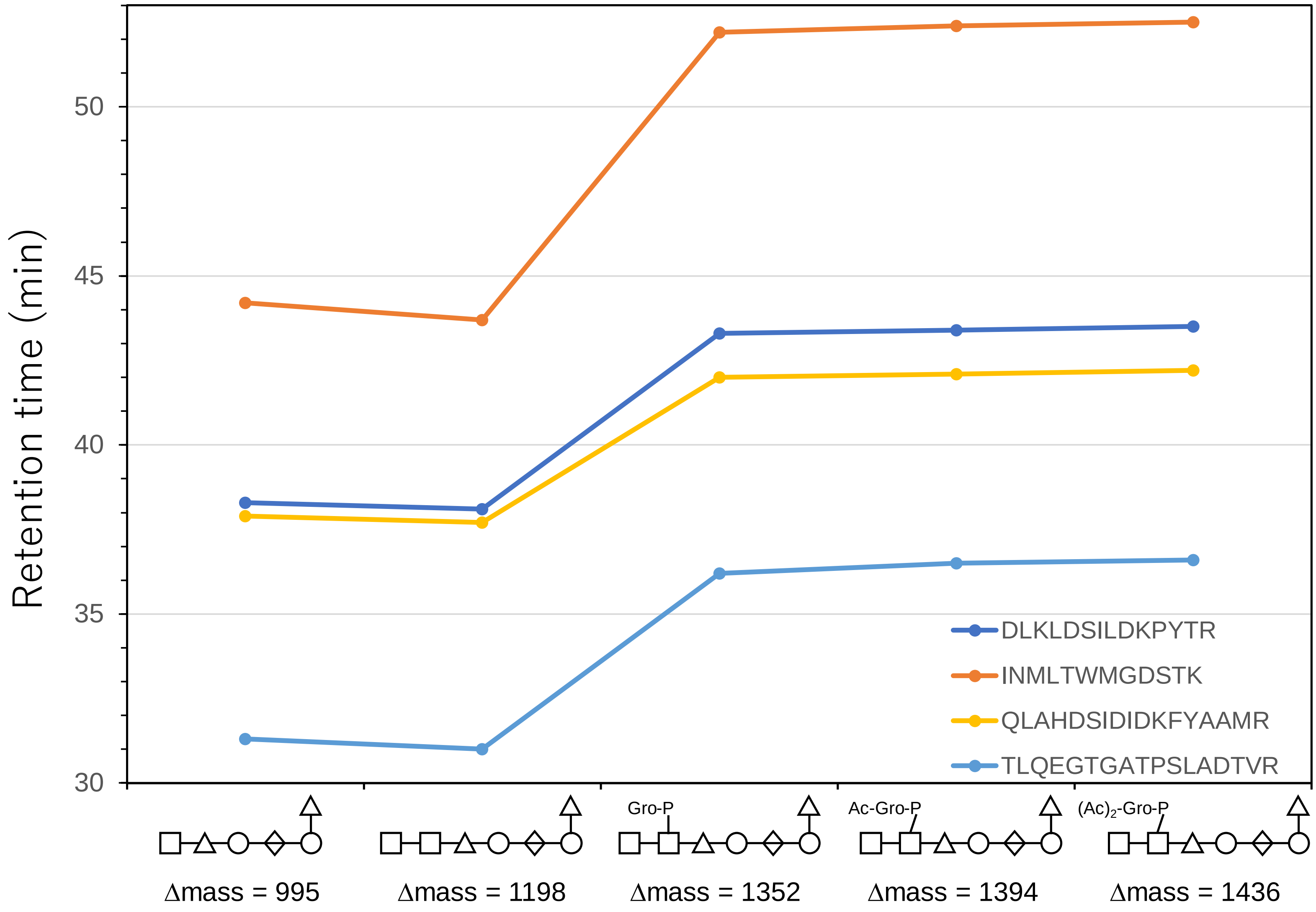

Figure S2. Heterogeneity of glycans with respect to phosphoglycerol. Mascot searches were repeated to identify glycopeptides lacking phosphoglycerol at Δmass values of 995 and 1198 Da. The HPLC retention times of these glycopeptides were then compared with their counterpart peptides substituted with phosphoglycerol. Data for four different peptides are provided as shown.

TABLE S1. Table of strains and primers

Strains used in this study.

Strains	Description	Source or reference
P. gingivalis		
ATCC 33277	Wild type	ATCC
KDP203	PGN_1302 (*waaL*)::Em^r	Shoji et al. 2011
KDP208	PGN_1033 (*wzx*)::Em^r	Shoji et al. 2013
KDP212	PGN_1523-1525::Em^r	Shoji et al. 2013
KDP213	PGN_1362-1363::Em^r	Shoji et al. 2013
KDP214	PGN_1896 (*wbaP*)::Em^r	Shoji et al. 2013
KDP604	PGN_0232-0234::Em^r	Shoji et al. 2018
KDP606	PGN_0428::Em^r	Shoji et al. 2018
KDP607	PGN_1134-1135::Em^r	Shoji et al. 2018
KDP610	PGN_1627-1628::Em^r	Shoji et al. 2018
KDP612	PGN_1724-1725::Em^r	Shoji et al. 2018
KDP623	PGN_1054 (*vimF*)::Em^r	Shoji et al. 2018
KDP1106	PGN_1010::Em^r	This study
KDP1107	PGN_1134::Em^r	This study
KDP1108	PGN_1135::Em^r	This study
KDP1109	PGN_1134::Em^r/pTCB-PGN_1134	This study
KDP1110	PGN_1135::Em^r/pTCB-PGN_1135	This study
E. coli		
XL-1 blue	Host strain for general purpose cloning	Stratagene

DNA primers used in this study.

Primer	Oligonucleotides (5'-)	Description
PGN1614upFw	GCATGCTGATCAATATGCTTACCGGCCAA	Underline indicates SphI site
PGN1614upRv	GGATCCTTCGGTTTCTTATTTCGTAGCTA	Underline indicates BamHI site
PGN1614dwFw	CTGCAGTCCGCAGCCCATTAACGCACCCG	Underline indicates PstI site
PGN1614dwRv	GAGCTCGTGGTGTGATAAATCGGAGTCAG	Underline indicates SacI site
PGN1010upFw	GATGCCATCATCAACCCGCTGATGGCACGA	
PGN1010upRv	CGGGGGTACCTATTGTCGATTATGAATAAG	
PGN1010dwFw	TAGGGGATCCACGAAACTCTTCAGTAAAAA	
PGN1010dwRv	CGGATGGGATACGATAATACCGGCGTTCTT	

ermF-Fw	ATCGACAATAGGTACCCCCGATAGCTTCCG	
ermF-Rv	AGAGTTTCGTGGATCCCCTACGAAGGATGA	
PGN1134dwFw	CTGCAGGCGATTCGTTTACCTCCCCATCGT	Underline indicates PstI site
PGN1134dwRv	GAGCTCGAGAGAGAGCATTCGGCAGCATGG	Underline indicates SacI site
PGN1135upFw	GCATGCCCATTTACTTTCGAACGAGCCATA	Underline indicates SphI site
PGN1135upRv	GGATCCCTCTCGGGCATGGTGGCAAATCGC	Underline indicates BamHI site
PGN1134compFw	GTCGACATGAATCCGCCCAAAAGACTGCTTGTCATT	Underline indicates SalI site
PGN1134compRv	TCTAGATTATCGGCTAAGCAGGGATAGCACAGGGGC	Underline indicates XbaI site
PGN1135compFw	GTCGACATGAAAATTCTCCTGCTGAATACATCCGAT	Underline indicates SalI site
PGN1135compRv	TCTAGATTAGAGCAGTTCGCGGTAGAGCTGCAGCAT	Underline indicates XbaI site

SUPPLEMENTARY REFERENCES

Shoji M, Sato K, Yukitake H, Kamaguchi A, Sasaki Y, Naito M, Nakayama K. 2018. Identification of genes encoding glycosyltransferases involved in lipopolysaccharide synthesis in *Porphyromonas gingivalis*. *Mol Oral Microbiol* 33:68-80.

Shoji M, Sato K, Yukitake H, Kondo Y, Narita Y, Kadowaki T, Naito M, Nakayama K. 2011. Por secretion system-dependent secretion and glycosylation of *Porphyromonas gingivalis* hemin-binding protein 35. *PLoS One* 6:e21372.

Shoji M, Yukitake H, Sato K, Shibata Y, Naito M, Aduse-Opoku J, Abiko Y, Curtis MA, Nakayama K. 2013. Identification of an O-antigen chain length regulator, WzzP, in *Porphyromonas gingivalis*. *Microbiologyopen* 2:383-401.

Veith PD, Gorasia DG, Reynolds EC. 2021. Towards defining the outer membrane proteome of *Porphyromonas gingivalis*. *Mol Oral Microbiol* 36:25-36.
